# Integrating Wearable Sensor Data With an AI-Based, Protocol-Flexible Triage Platform to Accelerate Decision-Making During the Golden Hour of Combat Casualty Care

**DOI:** 10.7759/cureus.91121

**Published:** 2025-08-27

**Authors:** Maitha K Alnuaimi

**Affiliations:** 1 General Practitioner, Zayed Military Hospital, Abu Dhabi, ARE

**Keywords:** artificial intelligence, combat casualty care, golden hour, interoperability, triage protocols, wearable sensors

## Abstract

Introduction

Rapid, accurate triage within the first “golden hour” on the battlefield is critical to survival, yet existing wearable sensors and manual protocols operate in isolation, delaying care and creating inconsistent priority ratings across military and civilian systems. We therefore designed a vendor-agnostic, protocol-flexible platform that ingests live biometric streams from military-grade wearables, applies an artificial intelligence (AI)-enhanced hybrid decision engine, and returns colour-coded casualty categories under any standard (e.g., MARCH (Massive hemorrhage, Airway, Respiration, Circulation, and Hypothermia/Head injury), Canadian Triage and Acuity Scale (CTAS), START(Screening Tool to Alert to the Right Treatment), SALT (Sort, Assess, Life-saving Interventions, Treatment/Transport)) in real time.

Methods

The planned platform will comprise four layers: (i) Data Ingestion, which normalizes ECG, heart-rate, blood-pressure, and oxygen saturation (SpO₂) signals to Open mHealth/Fast Healthcare Interoperability Resources (FHIR) schemas via BLE (Bluetooth Low Energy) or WebSockets; (ii) Secure Transport, which encrypts packets with Transport Layer Security (TLS) 1.3 and uses store-and-forward buffering through message queuing telemetry transport (MQTT)/Kafka brokers; (iii) Triage Engine, which merges continuous machine-learning severity scores with dynamically loaded guideline definitions (JSON (JavaScript Object Notation)/YAML (YAML Ain't Markup Language)) and rule-based overrides; and (iv) Presentation, which delivers one-tap guideline selection and actionable prompts to medics while providing a geo-tagged command dashboard. Validation will combine bench tests on synthetic and de-identified Medical Information Mart for Intensive Care IV (MIMIC-IV) datasets with randomized field-simulation trials that compare manual versus AI-augmented triage, measuring time to triage, protocol concordance, and medic cognitive load (National Aeronautics and Space Administration Task Load Index(NASA-TLX)).

Results (projected)

We anticipate at least a 30% reduction in triage time, agreement rates of 85% or higher with expert assessments across multiple protocols, and significant decreases in medic workload.

Conclusion

A dynamic, standards-based artificial intelligence (AI) triage platform could harmonize military and civilian casualty care, accelerate golden-hour decision-making, and improve multinational interoperability. Planned live-exercise evaluations and open-source protocol libraries are expected to facilitate rapid adoption and continuous refinement.

## Introduction

Timely intervention during the “golden hour” following battlefield injury is critical: analyses of United States combat fatalities from 2001 to 2011 revealed that 87.3% of deaths occurred before casualties reached a medical treatment facility, and nearly one‑quarter of those were deemed potentially survivable with optimized prehospital care [1]. In 2009, a Department of Defense policy mandating evacuation of critically injured service members within 60 minutes was associated with a significant decrease in case‑fatality rates, underscoring the life‑saving impact of accelerated casualty transport and care [2].

Although military‑issued wearable sensors, such as smartwatches capable of continuously monitoring electrocardiogram (ECG), heart rate, blood pressure, and oxygen saturation, have been field‑tested and commercially deployed, these devices currently function in isolation and require manual data interpretation by medics under fire [3]. Wearable sensors alone are insufficient in prehospital and combat environments, as they often suffer from poor data quality due to environmental and motion artifacts, lack interoperability standards, depend on reliable connectivity for real‑time use, and typically lack embedded decision‑support logic, compromising timely and accurate triage under stress [4]. Similarly, civilian studies have demonstrated the feasibility of severity‑scoring algorithms adapted to real‑time wearable streams, but none integrate seamlessly into combat environments or reflect military‑specific triage workflows [5].

Despite these technological advances, there is no unified platform that ingests heterogeneous wearable data, applies standardized or custom triage protocols, and delivers colour‑coded prioritization to both frontline providers and command elements in real time. Prehospital decision‑support reviews emphasize that sensor data alone are insufficient under austere conditions and must be coupled with robust clinical‑decision engines to guide non‑specialist responders [6].

Multiple triage schemas coexist in military and civilian practice: the United States military employs protocols such as the Massive Hemorrhage, Airway, Respiration, Circulation, Hypothermia (MARCH) algorithm and tactical combat casualty care guidelines (TCCC), mass‑casualty frameworks like Simple Triage and Rapid Treatment (START) and Sort, Assess, Life‑saving interventions, Treatment/Transport (SALT), and international systems such as the Canadian Triage and Acuity Scale (CTAS) [7-9]. By ‘modular decision engine,’ we refer to a software design in which independent, interchangeable components, e.g., for data parsing, severity scoring, or protocol mapping, can be updated or replaced without modifying the entire system. ‘Artificial intelligence (AI)‑driven’ means that machine‑learning models process biometric and operational data to produce continuous severity scores, which are then safeguarded by rule‑based overrides for clinical reliability. A truly adaptable platform must therefore support dynamic loading of any colour‑coded guideline, enabling interoperability across joint, coalition, and civilian disaster responses.

We thus propose the design of a vendor-agnostic, protocol-flexible AI-driven triage platform that, by standardizing wearable input via Open mHealth/FHIR schemas, securing data transport, and executing a modular decision engine, enables real-time, colour-coded casualty prioritization under any established or evolving triage guideline, while also accommodating supplementary clinical data when available.

## Materials and methods

Triage protocol

This is a conceptual and design paper outlining the architecture and planned capabilities of a vendor-agnostic, protocol-flexible system. The planned platform will support established military and civilian triage schemas, including the TCCC, MARCH algorithm widely used by United States forces [10], the CTAS for both civilian and expeditionary contexts [9], as well as mass casualty methods such as START and SALT developed for unplanned incidents [8]. To accommodate these diverse guidelines, the AI‑powered triage engine, built atop the OpenAI platform (OpenAI, Inc., San Francisco, California, United States) for scalable model management, must allow dynamic parameterization of vital‑sign thresholds, colour‑coding schemes, and category definitions via editable JSON (JavaScript Object Notation) and YAML (YAML Ain’t Markup Language) files, lightweight, human‑readable data‑serialization formats, ensuring that medics can load or update any protocol in real time without code changes.

Because the same incoming data stream can be reinterpreted under multiple schemas simultaneously, the system will let joint teams compare outputs side-by-side or switch instantly when a casualty moves from a military to a civilian chain of care. Moreover, its modular interface will also accept future score-based triage systems (e.g., National Early Warning Score 2 (NEWS2), Modified Early Warning Score (MEWS)) or specialty-specific guidelines without refactoring the core engine, highlighting true protocol-agnostic flexibility. In short, the AI-driven platform will be engineered for complex, resource-limited, and contested environments and deliver extreme adaptability and interoperability across diverse medical protocols and operational contexts.

User workflows

From the medic’s perspective, the mobile application must minimize cognitive load and manual input under fire by offering a “one‑tap” selector for the active triage guideline, after which incoming wearable data are automatically processed and displayed with a single, clear colour badge. Contextual prompts (e.g., “Control hemorrhage,” “Assess airway”) will guide immediate actions based on the chosen protocol, while secure voice/video overlay will be invoked with a simple swipe if remote consultation is required. On the command side, a web‑based dashboard will aggregate individual casualty statuses, regardless of protocol differences, into a unified operational map, providing real‑time situational awareness, resource‑preparation alerts, and an at‑a‑glance view of triage distributions to inform mass‑casualty response decisions [6].

In addition to the core triage workflows described above, the platform will incorporate the following advanced capabilities to enhance remote support, data integrity, and mass-casualty handling under austere conditions.

Secure Audio/Video Consultations

A low-latency video-call channel will allow medics or remote medical specialists to engage directly with patients or first-aid teams. This feature will support spoken guidance for advanced interventions, psychological support in cases where evacuation is impossible (e.g., hostile military zones), and remote mentoring of non-medical personnel. Video feeds will be encrypted end-to-end and can be toggled on demand within the Medic App.

Physiological Data Validation

The engine will continuously verify incoming biometric values against logical constraints (e.g., 0 ≤ SpO₂ ≤ 100 %). Implausible readings (e.g., SpO₂ = 500) will trigger flagging routines that request re-capture or sensor recalibration, ensuring that spurious data do not compromise triage decisions.

Contextual Activity Filtering

To reduce false alarms, the system will analyze minimal vital sign fluctuations in the context of known exertional events (e.g., running uphill, extreme heat). Activity metadata (accelerometer/BMI/exertion estimates) will be compared against individualized soldier profiles (including age, BMI, known health conditions, and blood type), enabling the engine to distinguish physiologic stress from pathologic deterioration.

Dynamic Case Grouping and Mass-Casualty Management

When multiple casualties are reported simultaneously, the dashboard will automatically cluster cases by triage category and location. Group-based summaries will highlight aggregate statistics (e.g., number of Reds, Yellows, Greens) and spawn templated alerts to designated medical teams (e.g., surgical, evacuation), optimizing resource allocation during mass-casualty incidents.

Geolocation and Estimated Time of Arrival (ETA)

Each device will embed Global Positioning System (GPS) coordinates within transmitted packets. The command dashboard will compute real-time distances between medic units and patient locations, provide ETA metrics, and factor in terrain and transit mode. This spatial intelligence will enable pre-positioning of evacuation assets and anticipatory care planning.

Energy Considerations

Because the triage engine is purely software, it will add virtually no additional power draw beyond the medic’s existing smartphone or tablet. All electrical demand will be concentrated in the wearable sensors themselves; short internal leads will carry only low‑voltage signals supplied by the sensors’ integrated rechargeable batteries, which are rated for at least 24 hours of continuous streaming. No extra wiring or external battery packs would be required, ensuring energy efficiency and simplifying field logistics.

Platform architecture

The proposed platform will be structured into four primary layers, each responsible for a distinct aspect of data handling, processing, and presentation. The end‑to‑end workflow, from wearable sensor activation to actionable output for medics and command, is described below.

Data Ingestion Layer

**Figure 1:**
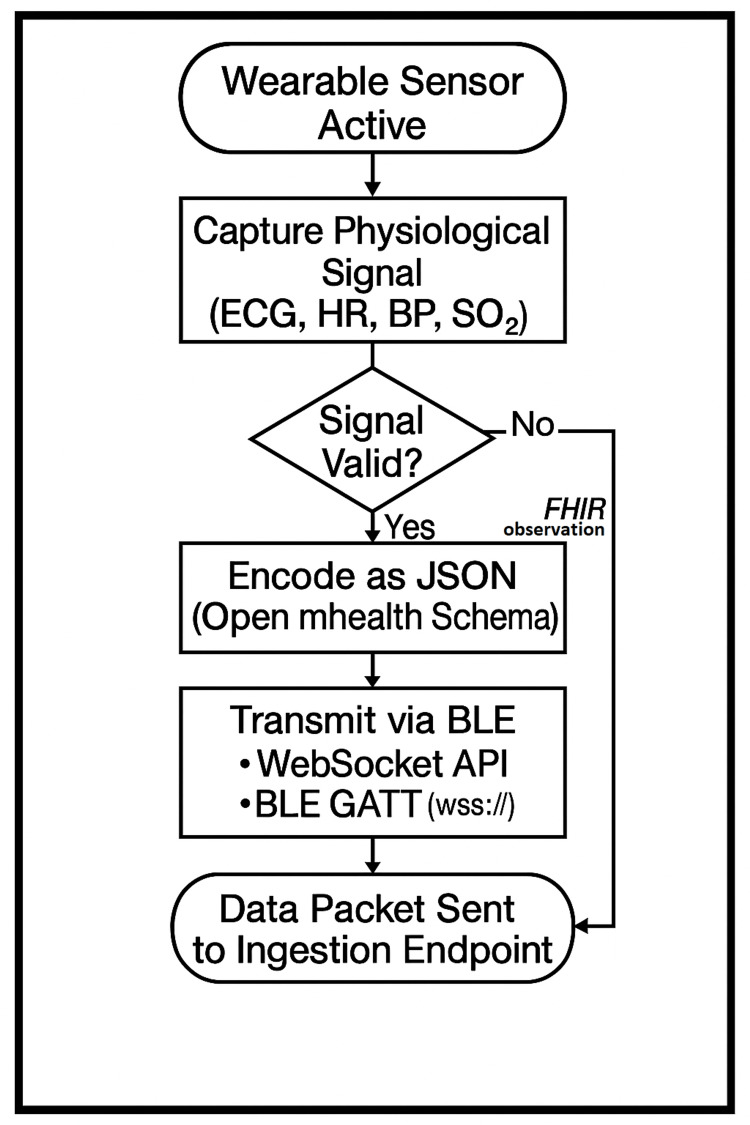
Workflow from Wearable Sensor Activation to Platform Ingestion Endpoint HR; heart rate; BP: blood pressure; SO2: oxygen saturation; FHIR: Fast Healthcare Interoperability Resources; JSON: JavaScript Object Notation; BLE: Bluetooth Low Energy; API: application programming interface; GATT: Generic Attribute Profile

This layer is planned to interface directly with existing wearable devices, leveraging vendor‑provided application programming interfaces (APIs) or software development kits (SDKs) (e.g., BLE (Bluetooth Low Energy) GATT (Generic Attribute Profile) profiles or secure WebSocket endpoints) to collect physiological signals in real time. Incoming packets would be normalized to an Open mHealth (London, United Kingdom) JSON schema and mapped to FHIR® (Fast Healthcare Interoperability Resources) observation resources to ensure interoperability with downstream systems. As illustrated in Figure 1, the workflow will begin when a wearable sensor activates and streams raw vitals to the platform’s ingestion endpoint.

Secure Transport Layer

**Figure 2:**
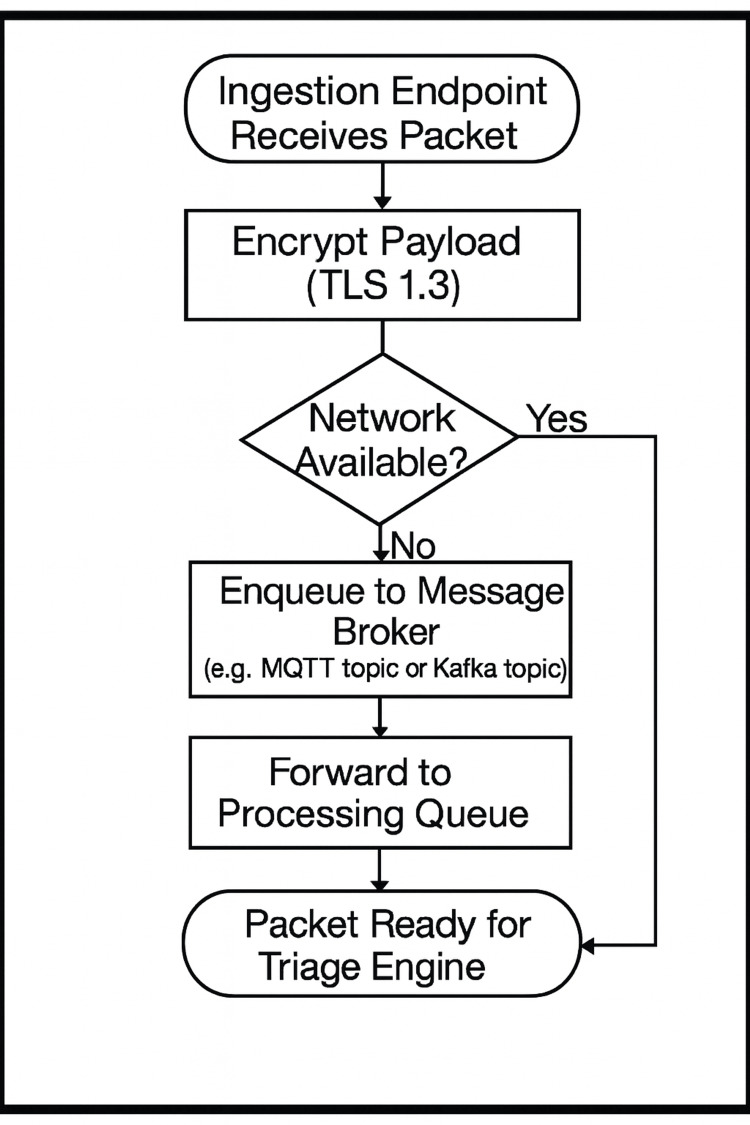
Transport & Queueing MQTT: message queuing telemetry transport; TLS: transport layer security

Once ingested, data packets would be encrypted using Transport Layer Security (TLS) 1.3 and subjected to a store‑and‑forward mechanism that buffers information locally during network outages. When connectivity is re‑established, queued packets would be published to a message broker (e.g., message queuing telemetry transport (MQTT) topic or Kafka topic) before being dispatched to the processing queue (Figure 2).

Triage Engine

**Figure 3:**
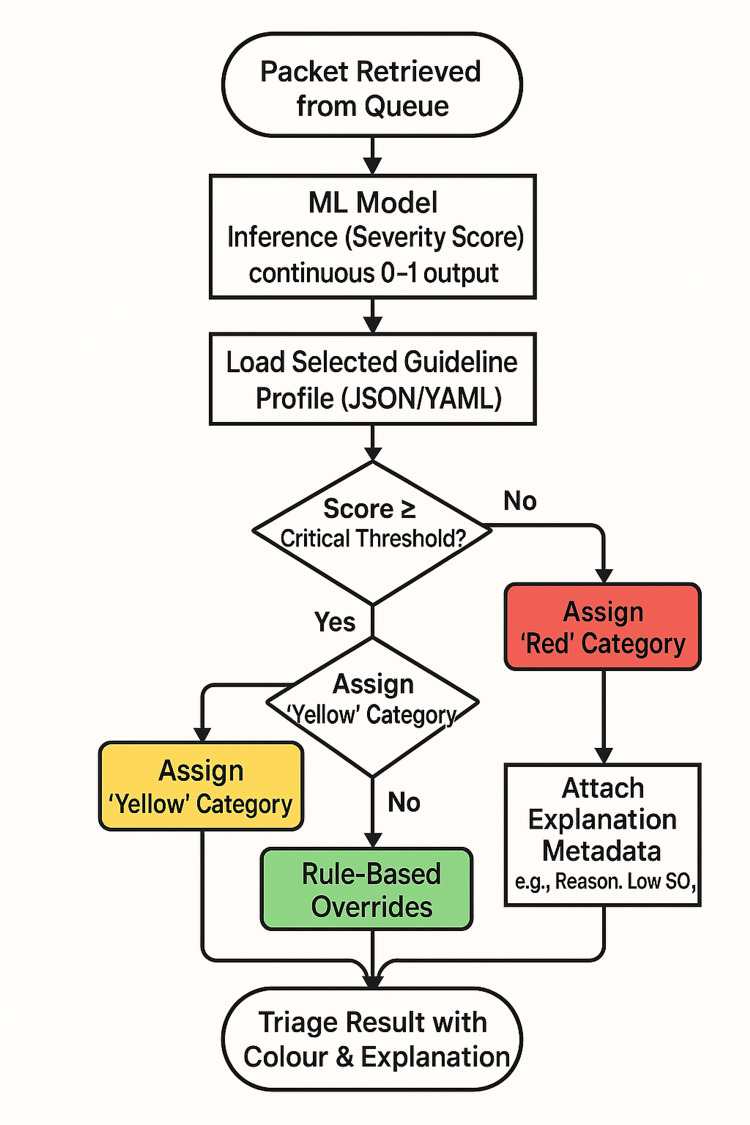
Processing and Decision JSON: JavaScript Object Notation; YAML: YAML Ain't Markup Language

At the core of the platform is a hybrid AI/rule‑based decision engine. First, the engine will parse JSON packets into feature vectors for model inference, producing a continuous severity score (0-1). Next, a dynamic guideline module will load the appropriate protocol definition (in JSON or YAML format), mapping scores and vital signs to colour‑coded categories (e.g., Red, Yellow, Green). Explainability hooks will annotate each decision with metadata (e.g., “Reason: Low SpO₂”), and rule‑based overrides (e.g., force Red if SpO₂ < 85 %) will ensure critical cases are never missed. The hybrid AI/rule‑based processing pipeline that will convert feature vectors into colour‑coded priorities is summarised in Figure 3.

Presentation Layer

**Figure 4:**
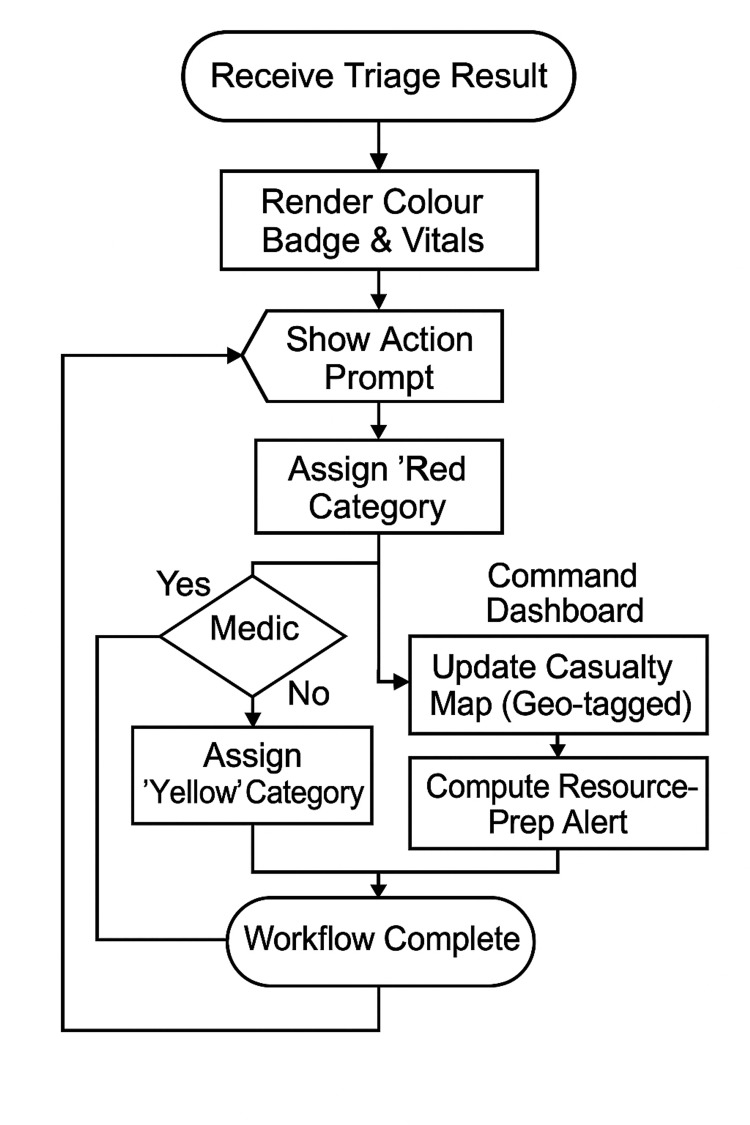
Display & Alerting

The final layer will deliver actionable insights to end users via two interfaces. The medic mobile application will display a real‑time colour badge and vital‑sign summary, along with context‑sensitive prompts (e.g., “Control hemorrhage”). A parallel command dashboard will aggregate geo‑tagged casualty statuses, compute resource‑preparation alerts, and issue notifications to command elements. The step‑by‑step workflow that will links the medic’s colour‑badge display to the command dashboard update is illustrated in Figure 4.

## Results

Validation strategy

To ensure the platform’s reliability and clinical relevance, we propose a three‑pronged validation approach covering bench testing, field simulation, and end‑user usability.

Bench Validation

The engine will be evaluated offline using synthetic trauma streams and de‑identified waveform data from Medical Information Mart for Intensive Care IV (MIMIC-IV) [11]. Multiple triage protocols (e.g., MARCH, START, CTAS) will be applied in parallel to the same dataset to assess three key dimensions:

(i) Latency: Time from packet ingestion to category assignment, targeting ≤500 ms per case.

(ii) Mapping accuracy: Proportion of cases correctly classified according to expert-labeled benchmarks (>90%).

(iii) Override handling: Verification that rule-based overrides (e.g., low SpO₂) consistently elevate cases to the appropriate category.

Simulation Trials

Controlled exercises will compare conventional manual triage against AI‑augmented workflows in randomized military field drills. Units will be assigned to follow either the standard process or the platform’s guidance under varied protocols. The study’s primary endpoints comprise two measures. First, Time to Triage captures the interval from “patient presentation” to the final category communicated. Second, Concordance per Protocol represents the agreement rate between platform output and board-certified combat medic assessments (target ≥ 85 %).

Usability Testing

Using the National Aeronautics and Space Administration Task Load Index(NASA-TLX), we will measure cognitive workload across protocol switches and real‑time decision points [12]. Key metrics are twofold. First, Protocol-Switch Workflow Time will record the seconds required for a medic to select or update the active guideline. Second, Cognitive Load Scores, expressed as comparative TLX ratings for manual versus AI-augmented triage, will aim for significant reductions in both mental demand and temporal demand.

## Discussion

A protocol‑agnostic design offers significant advantages for both military and civilian operations. By enabling the dynamic loading of any colour‑coded triage guideline, the platform facilitates smooth military‑to‑civilian handoff, ensuring that casualties transferred from battlefield care to civilian emergency departments encounter a consistent prioritization scheme [8]. Likewise, coalition interoperability is enhanced, and multinational forces can operate under their respective national protocols (e.g., CTAS in Canada vs. MARCH in the United States) without requiring separate systems or manual reclassification [9].

Recent evidence reinforces these advantages. An integrative systematic review of AI‑enabled emergency‑department triage systems found that machine‑learning models consistently outperformed traditional tools, achieving area‑under‑the‑curve values > 0.80 and reducing patient wait times by roughly 30% [13]. In parallel, a United States Military position paper emphasized that protocol‑flexible, vendor‑agnostic AI platforms are essential to modern operational medicine, delineating requirements that closely mirror our architecture [14].

However, this flexibility introduces new challenges. Maintaining guideline libraries demands a robust version‑control system to track updates, such as threshold changes or category re‑definitions, particularly when guidelines evolve during prolonged conflicts or after major public‑health events. Ensuring all deployed clients use the correct guideline version without human error requires automated distribution and consistency checks. Additionally, medic training must encompass not only device operation but also rapid switching between protocols under stress, which may increase cognitive load despite reduced manual calculations.

Compared to fixed‑protocol AI triage solutions developed for hospital settings (e.g., the Digital Triage Assistant, which hard‑codes EMS triage rules) [15] or APPRAISE-Hemorrhage Risk Index (HRI)’s hemorrhage‑risk model tailored to specific injury patterns [16], our platform’s hybrid engine-combining machine‑learning inference with rule‑based overrides, strikes a balance between adaptability and clinical safety. Hospital systems typically undergo extensive validation for a single protocol; in contrast, this framework must demonstrate equivalent reliability across a spectrum of schemas without re‑engineering the core inference pipeline.

Limitations

This prototype has yet to undergo real‑world testing and validation. Cloud‑hosted inference introduces a dependency on connectivity and data‑sovereignty rules that may require on‑prem alternatives for some deployments. Projected time‑savings are based on simulation drills and must be confirmed in live exercises. Finally, regulatory clearance pathways for algorithm‑guided triage are outside the scope of this paper and will be addressed in future work.

Additionally, certain technical limitations remain. Occasional data loss may occur during network outages despite buffering, and variability in sensor calibration can affect vital-sign accuracy. Furthermore, errors in uploaded JSON/YAML guideline files may disrupt triage outputs, underscoring the need for input validation and robust error handling.

Future directions

Future work will focus on three complementary tracks: conducting live joint‑forces exercises that place the platform in multi‑national field drills to verify cross‑protocol compatibility and command‑level situational awareness under real operational conditions; establishing an automated guideline‑update pipeline that ingests trusted feeds, such as WHO disaster‑triage revisions or updated Tactical Combat Casualty Care (TCCC) directives, and pushes new protocol definitions as machine‑readable JSON or YAML for seamless, policy‑driven adjustment; and publishing open‑source versions of core triage protocols under permissive licenses, together with a privacy‑safe “learning loop” that periodically retrains the severity‑scoring model on de‑identified field data, allowing the AI to refine its accuracy and decision thresholds after every deployment while inviting allied developers to audit rule logic, contribute enhancements, and help grow a shared ecosystem.

## Conclusions

By uniting vendor‐agnostic data ingestion, secure transport, a hybrid AI/rule‐based decision engine, and flexible presentation layers, this dynamic, standards‐based AI‐triage platform promises to transform combat casualty care. Its ability to load any established or emergent guideline ensures that medics and commanders operate on a shared priority schema-saving lives within the golden hour and simplifying multi‐nation operations across both military and civilian domains. In practice, this flexibility means new or updated protocols can simply be added as standalone JSON or YAML files, leaving the core software unchanged while keeping the system compatible with future standards.
